# Improved In-Hospital Outcomes for a Ruptured Abdominal Aortic Aneurysm Over Time: A Single-Center Retrospective Analysis of 58 Cases

**DOI:** 10.7759/cureus.101891

**Published:** 2026-01-20

**Authors:** Koki Yokawa, Taku Nakagawa, Makoto Kusakizako, Yosuke Tanaka, Tomonori Higuma, Kazunori Yoshida, Yoshihiro Oshima, Hidefumi Obo, Hidetaka Wakiyama

**Affiliations:** 1 Department of Cardiovascular Surgery, Kakogawa Central City Hospital, Kakogawa, JPN

**Keywords:** emergent surgery, endovascular therapy, management of abdominal aortic aneurysms, open surgery, ruptured abdominal aortic aneurysm

## Abstract

Objective: At our institution, open surgical repair (OS) is the first-line treatment for ruptured abdominal aortic aneurysms (rAAA). This study aimed to evaluate in-hospital and long-term outcomes of rAAA treatment in a community hospital setting and to assess temporal changes in management, including operative delay (time to surgery), and survival.

Methods: We retrospectively analyzed 58 patients (mean age: 74 ± 9 years; male: n = 47) who underwent emergency surgery for rAAA between November 2012 and March 2025. OS was performed as the primary treatment strategy in 51 cases, whereas EVAR was selectively performed in seven hemodynamically stable patients with suitable anatomy when an appropriate stent-graft device was available.

Results: In-hospital mortality rate was 13/58 (22.4%), all in the OS group. Deaths were due to multiple organ failure (n = 6), sepsis (n = 3), bowel necrosis (n = 2), and hemorrhage (n = 2). Mortality rate decreased from 37.9% (11/29) in the early group to 6.9% (2/29) in the late group, with shorter time to surgery (median: 109 vs. 217 minutes; p = 0.01). The five-year survival rate was 58% ± 7%.

Conclusion: A shorter time to surgery contributed to improved outcomes in patients with rAAA. Even when OS is adopted as the primary strategy, rapid surgical intervention can lead to favorable outcomes.

## Introduction

A ruptured abdominal aortic aneurysm (rAAA) is a highly critical condition, with a reported surgical mortality rate ranging from 17.6% to 31.6% [1]. Emergency surgical intervention is mandatory, and prompt initiation and completion of treatment are essential to improve outcomes. Recent improvements in emergency medical systems and advances in perioperative management specific to rAAA have contributed to improved survival [2]. Current Japanese guidelines recommend endovascular aneurysm repair (EVAR) as the first-line treatment for rAAA in patients with anatomically suitable features for EVAR within the device instructions for use (IFU) based on preoperative computed tomography (CT) assessment [1].

However, the optimal treatment strategy for rAAA remains controversial and highly dependent on patient condition and institutional logistics. The Fitzgerald classification, which describes the extent of retroperitoneal and intraperitoneal hemorrhage, has been shown to correlate closely with preoperative hemodynamic status and prognosis. Recent multicenter and single-center studies have proposed treatment algorithms based on this classification. In a multicenter observational study from Japan, EVAR was associated with lower in-hospital mortality in patients with Fitzgerald class I or II, whereas outcomes of EVAR and open surgical repair (OS) were comparable in Fitzgerald class III or IV without shock. Importantly, EVAR was associated with extremely poor outcomes in patients with Fitzgerald class IV, in whom OS appeared to be more beneficial [3]. Similarly, an EVAR-first strategy focusing on the Fitzgerald classification demonstrated favorable short- and mid-term outcomes in patients with limited hematoma extension while highlighting hematoma-related complications, including venous thrombosis and abdominal compartment syndrome, in more advanced classes [4].

Despite these findings, the widespread implementation of EVAR for rAAA is limited in many community hospitals. Endovascular devices are not always immediately available, and treatment may be delayed by the need for CT, anatomical assessment, device selection, and procurement. During this waiting period, patients may experience further hemodynamic deterioration, particularly those with extensive retroperitoneal hematoma or preoperative shock. Consequently, many patients undergo surgery in a severely compromised physiological state.

Furthermore, patients with advanced Fitzgerald classes are at increased risk of postoperative abdominal compartment syndrome due to massive retroperitoneal hematoma and ongoing capillary leak. In such critically ill patients, open abdominal management (OAM) using temporary abdominal closure with negative pressure therapy has been adopted as an adjunctive strategy to control intra-abdominal pressure and facilitate staged abdominal closure.

Accordingly, our institution has adopted OS as the primary treatment strategy for rAAA, with selective use of OAM in high-risk patients. However, there remains a paucity of evidence regarding outcomes and time-dependent improvements in rAAA management in community hospitals where immediate EVAR availability cannot be guaranteed, and rapid surgical control is prioritized. Therefore, this study aimed to report our real-world outcomes with an OS-first strategy, evaluate temporal changes in preoperative waiting time and survival, and explore practical management considerations applicable to resource-limited settings.

## Materials and methods

Patients

**Figure 1:**
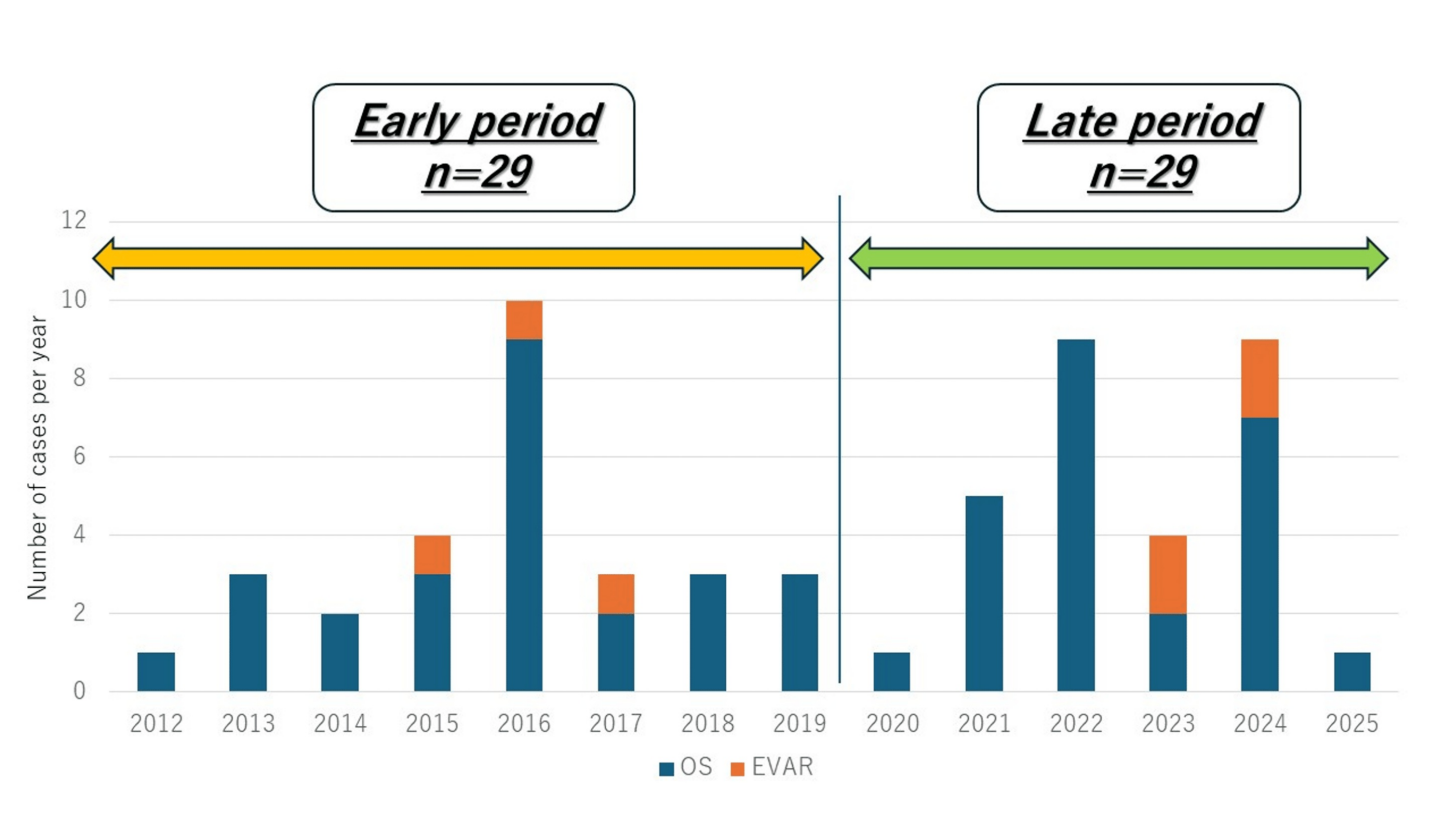
Annual number of surgeries for ruptured abdominal aortic aneurysm (rAAA). Annual number of surgeries for ruptured abdominal aortic aneurysm (rAAA). Blue bars represent open surgical repair (OS), and orange bars represent endovascular aneurysm repair (EVAR).

In this study, 58 patients with rAAA who underwent emergency surgery between November 2012 and March 2025 at our institution were analyzed retrospectively. We included consecutive patients who were transported to our institution and underwent emergency surgical repair for ruptured abdominal aortic aneurysm during the study period. Patients who did not undergo surgical repair at our institution were not included. The mean patient age was 74 ± 9 years, and 47 (81%) were male. Among the patients, 24 presented with preoperative hemodynamic shock, and three experienced cardiopulmonary arrest before the surgery started. The inferior vena cava was perforated in three patients and the intestines in one following aneurysm rupture. The patient cohort was divided into two time-based groups: an early period (2012-2019, n = 29) and a late period (2020-2025, n = 29). Treatment outcomes were evaluated between these groups. Figure 1 illustrates the number of cases during these periods.

Fitzgerald classification

Ruptured abdominal aortic aneurysms were classified according to the Fitzgerald classification, which categorizes patients into four groups based on the extent of retroperitoneal hematoma and the presence of free intraperitoneal rupture [3]. Group I is characterized by a small retroperitoneal hematoma confined to the site of rupture. Group II includes cases with a retroperitoneal hematoma extending into the pelvis. Group III is defined by a large retroperitoneal hematoma extending above the renal arteries or crossing the midline. Group IV represents free intraperitoneal rupture accompanied by diffuse intraperitoneal hemorrhage. The Fitzgerald classification was applied to assess the extent of rupture on preoperative CT, revealing type I in five cases, type II in 10 cases, type III in 19 cases, and type IV in 24 cases.

Operative procedure

Open surgical repair was the primary treatment strategy at our institution. EVAR was selectively performed in hemodynamically stable patients with anatomically suitable features within the device IFU when an appropriate stent-graft device was available. Accordingly, EVAR could only be performed in seven patients who had stable hemodynamics, and OS was performed in the remaining 51 patients. Among patients with unstable hemodynamics, four patients underwent intra-aortic balloon occlusion (IABO) preoperatively. In cases suggestive of abdominal compartment syndrome - particularly in open surgery cases with severe intestinal edema - our surgical strategy favored planned open abdomen management with delayed primary closure. Since May 2023, we have used the ABTHERA™ system for temporary abdominal closure and postoperative management. OAM was performed using the ABTHERA™ Open Abdomen Negative Pressure Therapy System (3M, St. Paul, MN), which has been reported to improve primary fascial closure rates compared with conventional temporary abdominal closure techniques [4]. Perioperative management was provided according to institutional emergency pathways, including rapid transfer to the operating room for definitive hemorrhage control. In selected high-risk patients, OAM with delayed abdominal closure was performed to mitigate postoperative abdominal compartment syndrome.

Perioperative time and group comparison

The operative waiting time referred to the interval between patient arrival and surgical initiation. Yearly trends in the waiting time were illustrated using box plots and median values with interquartile ranges. To evaluate the potential relationship between the time to surgery and in-hospital mortality, the time interval from hospital arrival to skin incision was compared between the early and late groups. EVAR cases were excluded from this analysis, as the need to order devices after diagnosis prolongs the preoperative period, which does not reflect institutional readiness or logistical improvements.

Data collection and statistical analysis

This retrospective observational study was conducted at Kakogawa Central City Hospital, a community hospital in Hyogo, Japan. Data were collected from outpatient and hospital admission medical records. Continuous variables are expressed as the mean ± standard deviation or median (interquartile range: 25th-75th percentiles), as appropriate. Categorical variables are presented as counts and percentages.

Continuous variables were compared using Student's t-test, and categorical variables were compared using the chi-square test or Fisher's exact test, as appropriate. Spearman's rank correlation coefficient was calculated to assess the association between the calendar year and the waiting time to surgery. Linear regression analysis was also performed to evaluate temporal trends in waiting time.

All statistical analyses were conducted using Statistical Product and Service Solutions (SPSS, IBM SPSS Statistics for Windows, Armonk, NY). The Kaplan-Meier curve is presented descriptively to illustrate overall survival trends, without statistical comparison between groups. Postoperative follow-up data were obtained from outpatient medical records at our institution. Survival status was assessed based on available clinical documentation during the follow-up period. A p value of <0.05 was considered statistically significant.

The need for oral and written informed consent was waived owing to the retrospective nature of this study. This observational study was approved by the institutional review board (approval no. 2024-35).

## Results

Table 1 presents the characteristics of the patients in the early and late groups. No significant differences in preoperative status were observed between the two groups. Figure 1 shows the number of surgeries performed for rAAA during the study period.

**Table 1:** Patients’ characteristics. CPA: cardiopulmonary arrest Data are presented as mean ± standard deviation or number (percentage), as appropriate. Continuous variables were compared using Student's t-test, and categorical variables were compared using the chi-square test or Fisher's exact test, as appropriate. Corresponding test statistics (t-value or χ² value) are shown. P values indicate comparisons between the early and late groups. Statistical comparison was not performed for variables with extremely small sample sizes.

Variables	All (n=58)	Early (n=29)	Late (n=29)	p value (early vs late)
Age (y.o)	74±9	73±9	75±9	0.54
Male	47	24 (82)	23 (79)	0.73
Preoperative shock	24	12 (41)	12 (41)	1
Preoperative CPA	3	0	3	NA
Fitzgerald classification				
I	5	3 (10)	2 (10)	0.64
II	10	4 (14)	6 (20)	0.48
III	19	6 (21)	13 (44)	0.05
IV	24	16 (55)	8 (26)	0.01

OS was performed in 51 patients, whereas EVAR was performed in seven patients. The in-hospital mortality rate was 22.4% (n = 13). The causes of death included multiorgan failure in six patients, bowel necrosis in two, sepsis in three, and hemorrhage in two. Among patients who died during hospitalization, three had Fitzgerald type III, and 10 had type IV. Twelve patients were in shock preoperatively, including three who experienced cardiopulmonary arrest. IABO was performed in four patients, whereas attempts were unsuccessful in three. Abdominal closure was delayed in 16 patients, and the findings are summarized in Table 2.

**Table 2:** Operative procedures for a ruptured abdominal aortic aneurysm. EVAR: endovascular aneurysm repair Data are presented as a number (percentage). Categorical variables were compared using the chi-square test or Fisher's exact test, as appropriate. Corresponding test statistics are shown. P values indicate comparisons between the early and late groups. Statistical comparison was not performed for variables with extremely small sample sizes.

Procedure	All	Early	Late	p value (early vs late)
Open surgery	51	26 (90)	25 (86)	0.69
EVAR	7	3 (10)	4 (14)	0.69
Intra-aortic balloon occlusion	4	4 (14)	0	NA
Open abdominal management	16	7 (24)	9 (31)	0.55

**Figure 2:**
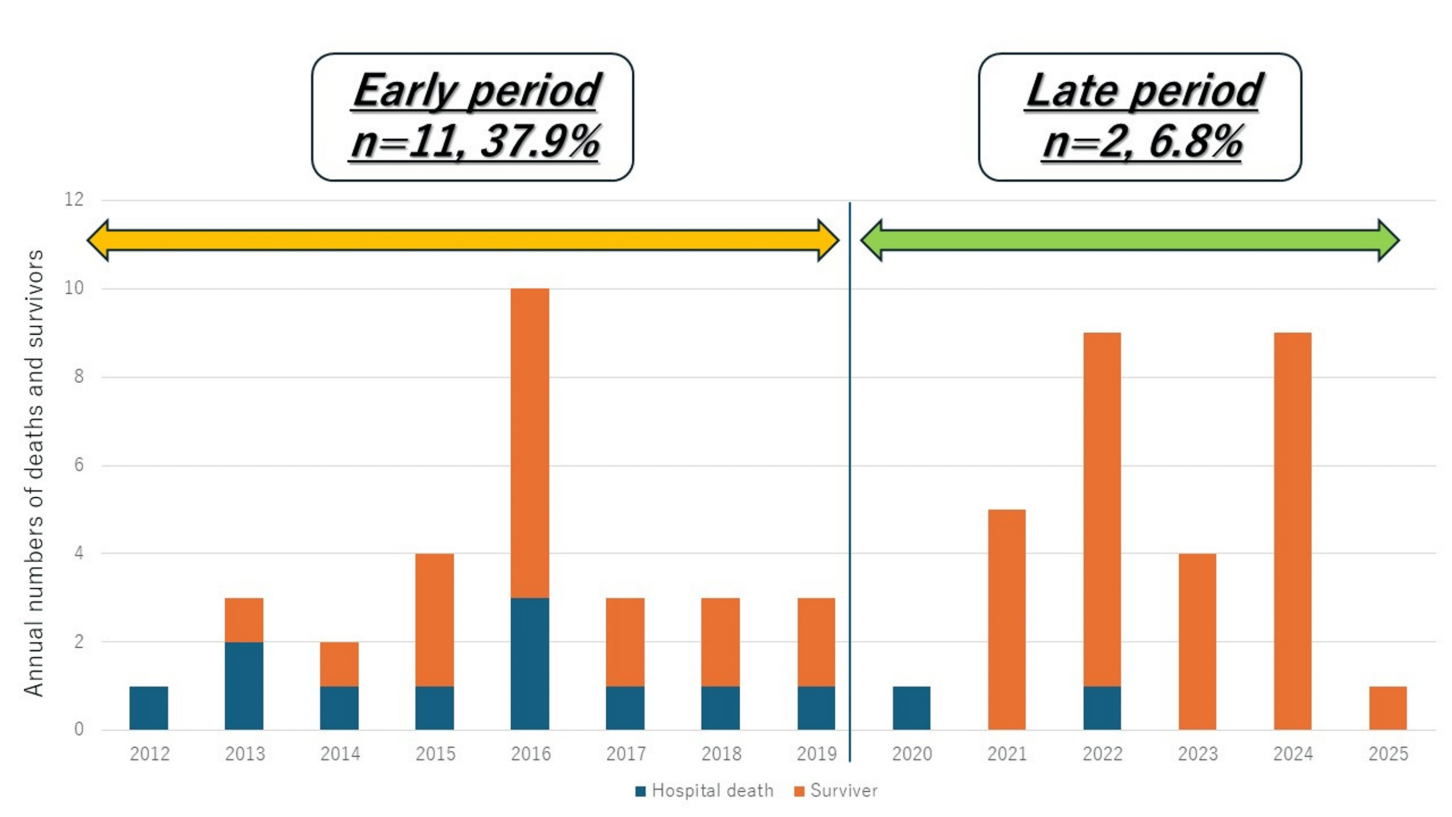
Annual numbers of deaths and survivors. Blue bars indicate deaths, and orange bars indicate survivors.

**Figure 3:**
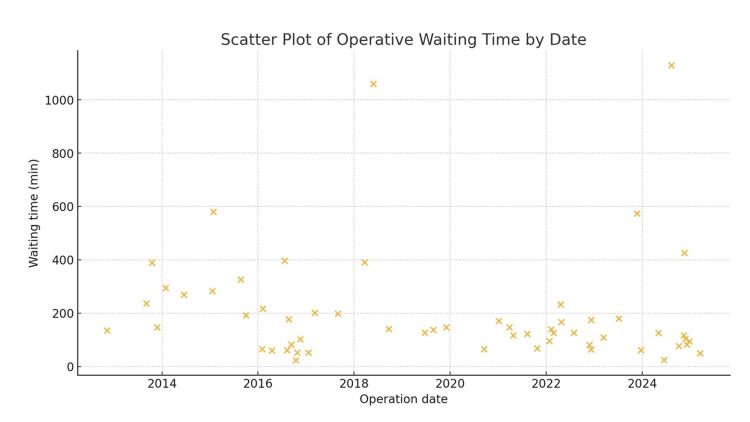
Yearly trend in operative waiting time. Scatter plot of the operative waiting time for each patient according to the operation date. Spearman's rank correlation analysis revealed a significant negative correlation between calendar year and waiting time (ρ = -0.28, p = 0.02).

In the early group, 11 in-hospital deaths (37.9%) occurred, whereas in the late group, only two (6.8%) deaths occurred. Figure 2 illustrates the yearly numbers of deaths and survivors during this period, revealing a significantly lower mortality in the late period (p = 0.002). The median time from hospital arrival to skin incision was significantly shortened in the late group compared with that in the early group (109 ± 46 vs. 217 ± 215 min, respectively; p = 0.01). The operative waiting time significantly shortened over the study period (Spearman's ρ = -0.28; p = 0.02). The yearly median waiting time shortened from 282 min in 2014 to 49 minutes in 2025 (Figure 3). EVAR cases were excluded from this analysis because of delays in device procurement. The marked reduction in operative delay in the late group may partly explain the observed improvement in in-hospital survival. However, this relationship should be interpreted with caution because other unmeasured confounders may also have contributed to the improved outcomes.

Regarding long-term outcomes, the five-year survival rate was 58% ± 7%. One patient who underwent EVAR experienced aneurysmal enlargement postoperatively. Endovascular reintervention was performed for a type Ia endoleak 54 months after the initial surgery, and open abdominal aortic replacement was conducted 104 months later due to further aneurysmal expansion.

## Discussion

Although current guidelines advocate for EVAR as the first-line treatment for rAAA, many community hospitals do not always have stent graft devices immediately available. Consequently, treatment selection is often constrained by institutional logistics rather than patient anatomy alone. In this context, our study provides valuable insights into the real-world challenges faced by community hospitals managing rAAA.

Seike et al. examined the outcomes of rAAA treated with OS as the primary strategy and demonstrated that surgical outcomes were not significantly influenced by the specific operative technique employed [5]. Their findings support the validity of an OS-first strategy in institutions where timely EVAR cannot be guaranteed. Furthermore, recent studies have emphasized the importance of patient stratification based on hemorrhage extent using the Fitzgerald classification. Multicenter data from Japan suggest that EVAR yields favorable outcomes in patients with Fitzgerald class I or II, whereas outcomes are comparable or even inferior to OS in patients with more advanced classes, particularly those with preoperative shock or Fitzgerald class IV [6,7]. These findings indicate that EVAR is not universally optimal and that OS remains an essential treatment option for patients with extensive retroperitoneal or intraperitoneal hemorrhage.

In patients with unstable hemodynamics, intra-aortic balloon occlusion (IABO) has been reported to be effective as a bridge to definitive repair [8]. However, this technique often requires rapid initiation in the emergency department and may be difficult to implement consistently, particularly in resource-limited settings. Accordingly, our institutional strategy prioritized rapid transfer to the operating room and immediate surgical control without reliance on preoperative IABO, thereby minimizing delays in definitive hemostasis.

Recently, advances in OAM, including the introduction of negative pressure therapy systems such as the ABTHERA™ system, have improved postoperative management in critically ill patients requiring delayed abdominal closure [4,9]. Our department has proactively adopted an OAM strategy in selected high-risk cases following OS. Notably, several patients who died from multiorgan failure or intestinal necrosis in the early postoperative period had undergone primary abdominal closure at the end of surgery. This observation suggests that avoiding primary closure and instead employing staged abdominal management may help mitigate abdominal compartment syndrome and improve postoperative physiological recovery in severely compromised patients.

Another important factor contributing to improved outcomes in the late group was the reduction in time from hospital arrival to surgical intervention. In July 2016, our institution underwent a major organizational integration that resulted in the establishment of a more efficient emergency surgical system. We previously reported improved outcomes in acute Stanford type A aortic dissection following this integration, with a significant reduction in time from symptom onset to surgery [10]. Consistent with these findings, the present study demonstrated shortened preoperative waiting times for rAAA. Enhanced institutional readiness and streamlined emergency workflows likely played a critical role in reducing in-hospital mortality, underscoring the importance of hospital-level systems in optimizing outcomes for rAAA.

This study has several limitations. First, its retrospective design may have introduced selection bias and limited our ability to control for confounding factors. Second, the sample size was relatively small, and the analysis was conducted at a single institution, which may limit the generalizability of our findings. Third, because this study included only patients who underwent emergency surgical repair at our hospital, we were unable to fully capture patients who were transferred to other institutions, managed non-operatively, or died before surgical intervention, which may have affected the representativeness of the study population. Fourth, EVAR cases were excluded from the waiting-time analysis because device procurement strongly influenced the preoperative interval; however, this exclusion may have introduced selection bias and reduced comparability between periods. Fifth, the Fitzgerald classification was assessed by a single reviewer (the first author) without evaluation of interobserver reliability and thus may be subject to observer bias. Finally, multiple surgeons were involved in operative management, which may have contributed to variability in intraoperative decision-making and outcomes. Despite these limitations, our findings underscore the continued importance of OS, rapid access to definitive hemostasis, and tailored postoperative management strategies, including open abdominal management, to optimize outcomes for rAAA in community hospital settings.

## Conclusions

OS as the primary strategy for rAAA achieved acceptable outcomes in a community hospital setting. While EVAR is recommended in current guidelines, its feasibility is limited by patient and institutional factors, and OS remains indispensable, particularly in advanced cases. Improved outcomes were associated with both optimized perioperative management and enhanced institutional readiness, emphasizing the importance of system-level factors in rAAA care.
